# Hydrogen sulphide increases pulmonary veins and atrial arrhythmogenesis with activation of protein kinase C

**DOI:** 10.1111/jcmm.13627

**Published:** 2018-04-16

**Authors:** Chao‐Shun Chan, Yung‐Kuo Lin, Yu‐Hsun Kao, Yao‐Chang Chen, Shih‐Ann Chen, Yi‐Jen Chen

**Affiliations:** ^1^ Graduate Institute of Clinical Medicine College of Medicine Taipei Medical University Taipei Taiwan; ^2^ Division of Cardiology Department of Internal Medicine Taipei Medical University Hospital Taipei Taiwan; ^3^ Division of Cardiology Department of Internal Medicine School of Medicine College of Medicine Taipei Medical University Taipei Taiwan; ^4^ Division of Cardiovascular Medicine Department of Internal Medicine Wang Fang Hospital Taipei Medical University Taipei Taiwan; ^5^ Department of Medical Education and Research Wan Fang Hospital Taipei Medical University Taipei Taiwan; ^6^ Department of Biomedical Engineering National Defense Medical Center Taipei Taiwan; ^7^ Division of Cardiology Department of Medicine Taipei Veterans General Hospital and Institute of Clinical Medicine and Cardiovascular Research Center National Yang‐Ming University Taipei Taiwan

**Keywords:** atrial fibrillation, hydrogen sulphide, protein kinase C

## Abstract

Hydrogen sulphide (H_2_S), one of the most common toxic air pollutants, is an important aetiology of atrial fibrillation (AF). Pulmonary veins (PVs) and left atrium (LA) are the most important AF trigger and substrate. We investigated whether H_2_S may modulate the arrhythmogenesis of PVs and atria. Conventional microelectrodes and whole‐cell patch clamp were performed in rabbit PV, sinoatrial node (SAN) or atrial cardiomyocytes before and after the perfusion of NaHS with or without chelerythrine (a selective PKC inhibitor), rottlerin (a specific PKC δ inhibitor) or KB‐R7943 (a NCX inhibitor). NaHS reduced spontaneous beating rates, but increased the occurrences of delayed afterdepolarizations and burst firing in PVs and SANs. NaHS (100 μmol/L) increased I_KATP_ and I_NCX_ in PV and LA cardiomyocytes, which were attenuated by chelerythrine (3 μmol/L). Chelerythrine, rottlerin (10 μmol/L) or KB‐R7943 (10 μmol/L) attenuated the arrhythmogenic effects of NaHS on PVs or SANs. NaHS shortened the action potential duration in LA, but not in right atrium or in the presence of chelerythrine. NaHS increased PKC activity, but did not translocate PKC isoforms α, ε to membrane in LA. In conclusion, through protein kinase C signalling, H_2_S increases PV and atrial arrhythmogenesis, which may contribute to air pollution‐induced AF.

## 1 INTRODUCTION

Atrial fibrillation (AF), the most common sustained cardiac arrhythmia, increases the incidences of heart failure, stroke and mortality.^1^, ^2^, ^3^, ^4^ Air pollution increases the risk of AF.^5^, ^6^ Each 6.0 μg/m^3^ increase in PM_2.5_ increases the risk of AF by 26%.^5^ The mechanisms underlying air pollution‐induced arrhythmogenesis remain unclear. Air pollution is associated with autonomic tone changes,^7^ inflammation^8^ and cardiac ischaemia.^9^ Hydrogen sulphide (H_2_S) is one of the most common toxic air pollutants.^10^ H_2_S is produced by the anaerobic bacterial breakdown of sulphur‐containing matter and can be found in various natural environments and industrial settings, such as spas, sewers, landfills, waste water plants and oil refineries.^11^, ^12^ Increases in the H_2_S concentration are associated with daily all‐natural‐cause mortality^13^ and cardiovascular hospitalization.^14^ However, it is not elucidated whether H_2_S may play a role in the pathophysiology of air pollution‐induced AF.

Pulmonary veins (PVs) and left atrium (LA) are the most important AF triggers and substrates.^15^, ^16^, ^17^ Calcium dysregulation plays a critical role in the occurrences of AF and PV arrhythmogenesis.^18^, ^19^ The activation of Na^+^/Ca^2+^ exchangers (NCXs) induces delayed afterdepolarizations (DADs) and increases PV arrhythmogenic activity.^18^ Protein kinase C (PKC)‐mediated signalling plays a vital role in NCX activation.^20^ H_2_S was known to activate PKC,^21^ thus H_2_S may increase I_NCX_ and increase PV arrhythmogenesis leading to AF genesis. Moreover, sinoatrial node (SAN) dysfunction plays an important role in AF pathophysiology^22^ and increases PV arrhythmogenesis.^23^ H_2_S reduces the electrical activity of SANs, which may modulate the arrhythmogenesis of PVs and increase the risk of AF genesis.^24^

H_2_S plays a critical role in cell signalling^25^ and attenuates ischaemia‐reperfusion injury by activating the ATP‐sensitive potassium channel (K_ATP_).^26^ The activation of the K_ATP_ channel shortens the action potential duration (APD), which may increase the risk of AF by facilitating the genesis of re‐entry circuits. Previous study has revealed that I_KATP_ differentially regulates the electrical activity of right atrium (RA) and LA.^27^ The different effects of I_KATP_ on APD shortening in the LA and RA can increase the risk of cardiac arrhythmias due to increasing interatrial dispersion. Accordingly, H_2_S may modulate the electrical activity of PVs, atria and SANs and increase the risk of air pollution‐induced AF. This study explored the effects of H_2_S on PVs, atria and SANs, and investigated the potential underlying mechanisms.

## 2 METHODS

### 2.1 Animal and tissue preparation

All experimental procedures were approved by the Institutional Animal Care and Use Committee of Taipei Medical University and conformed to the Institutional Guidelines for the Care and Use of Laboratory Animals and the Guide for the Care and Use of Laboratory Animals published by the United States National Institute of Health. As described previously,^28^ male rabbits (1.5‐2 kg) were anaesthetized with an intravenous injection of sodium pentobarbital (100 mg/kg). The adequacy of the anaesthesia was confirmed by the lack of corneal reflexes and motor responses to pain stimuli induced using a scalpel tip. The heart and lungs were rapidly excised following midline thoracotomy. For SAN tissue preparation, SANs were isolated from the RA and superior vena cava. PVs were separated from the atria at the LA‐PV junction and from the lungs at the end of PV myocardial sleeves. One end of the preparation was pinned to the bottom of a tissue bath using needles, and the other end was connected to a Grass Instruments FT03C force transducer (MA, USA) using silk thread. Tissue preparations were superfused with normal Tyrode's solution composed of NaCl (137 mmol/L), KCl (4 mmol/L), NaHCO_3_ (15 mmol/L), NaH_2_PO_4_ (0.5 mmol/L), MgCl_2_ (0.5 mmol/L), CaCl_2_ (2.7 mmol/L) and dextrose (11 mmol/L) at a constant rate of 3 mL/min at 37°C as described previously.^18^, ^19^, ^23^, ^27^ NaHS (Sigma, MO, USA) was used as a donor of H_2_S. PVs, atria and SANs were exposed to different concentrations of NaHS (1, 10, and 100 μmol/L) in Tyrode's solution for 40 minutes to investigate the electrophysiological effects of H_2_S.

### 2.2 Electrophysiological and pharmacological studies

The transmembrane APs of PVs, SANs and atria were recorded using machine‐pulled glass capillary microelectrodes filled with 3 mol/L KCl; the microelectrodes were connected to a World Precision Instrument model FD223 electrometer (FL, USA) under a tension of 150 mg. The electrical and mechanical events were simultaneously displayed on a Gould 4072 oscilloscope (OH, USA) and Gould TA11 recorder. Electrical stimuli were applied using a Grass S88 stimulator through a Grass SIU5B stimulus isolation unit. For PVs with spontaneous activity and SANs, the APs were recorded for 20 minutes. For the LA and RA, the AP parameters were measured with 2‐Hz electrical stimuli. The AP amplitude (APA) was determined by measuring the difference between the resting membrane potential (RMP) and the peak of AP depolarization. The APD at repolarization extents of 90%, 50% and 20% of the APD were measured and designated APD_90_, APD_50_ and APD_20,_ respectively. Burst firing was defined as the occurrence of accelerated spontaneous activities (faster than the basal rate) with sudden onset and termination. DAD was defined as the presence of a spontaneous depolarization of the impulse after complete repolarization. The electrical and mechanical events (contractility and diastolic tension) were continuously and simultaneously displayed and recorded during all aforementioned procedures. To investigate the electrophysiological effects of H_2_S, a physiological concentration of NaHS (100 μmol/L) was administered with or without KB‐R7943 (a NCX inhibitor, 10 μmol/L), chelerythrine (a selective PKC inhibitor, 3 μmol/L) or rottlerin (a specific PKC δ inhibitor, 10 μmol/L) in PVs, atria and SANs.

### 2.3 Electropharmacological studies in isolated single PV and atrial cardiomyocytes

Pulmonary vein and atria cardiomyocytes from rabbits were enzymatically dissociated, as previously described.^29^, ^30^ The whole‐cell patch clamp technique was performed in the PV and atrial cardiomyocytes with pacemaker activity before and after the administration of NaHS with or without chelerythrine; the APs were recorded using an Axopatch 1D amplifier (Axon Instruments, California, USA) at 35°C ± 1°C. The ionic currents were recorded in the voltage clamp mode. For the I_NCX_, PV and atrial cardiomyocytes were perfused with an external solution containing NaCl (140 mmol/L), CaCl_2_ (2 mmol/L), MgCl_2_ (1 mmol/L), glucose (10 mmol/L) and HEPES (5 mmol/L) (pH adjusted to 7.4 with NaOH/HCl). Micropipettes were filled with a solution containing NaCl (20 mmol/L), CsCl (110 mmol/L), MgCl_2_ (0.4 mmol/L), CaCl_2_ (1.75 mmol/L), TEACl (20 mmol/L), BAPTA (5 mmol/L), glucose (5 mmol/L), MgATP (5 mmol/L) and HEPES (10 mmol/L) (pH adjusted to 7.25 with CsOH). The I_NCX_ was elicited through depolarization in 10‐mV steps from a holding potential of −40 mV to test potentials from −100 to +100 mV for 300 mseconds at a frequency of 0.1 Hz. The I_NCX_ amplitudes were measured as 10‐mmol/L nickel‐sensitive currents. For the I_KATP_, PV and atria cardiomyocytes were perfused with an external solution containing NaCl (135 mmol/L), KCl (5.4 mmol/L), MgCl_2_ (1.0 mmol/L), CaCl_2_ (1.0 mmol/L), NaH_2_PO_4_ (0.33 mmol/L), HEPES (10 mmol/L) and glucose (10 mmol/L) (pH adjusted to 7.4 with NaOH). CdCl_2_ (0.2 mmol/L) and 4‐aminopyridine (2 mmol/L) were added to the external solution to inhibit Ca^2+^ and transient outward currents, respectively.^31^ Micropipettes were filled with a solution containing KCl (140 mmol/L), MgCl_2_ (1.0 mmol/L), HEPES (10 mmol/L), EGTA (5 mmol/L) and GTP (0.1 mmol/L) (pH adjusted to 7.3 with KOH).

### 2.4 Cell fractionation and Western blot for translocated PKC

Membrane and cytosol fractionations were performed from LA tissues using Mem‐PER Plus Membrane Protein Extraction Kit (Thermo Scientific, Waltham, MA) according manufacturer's instructions. Briefly, LA tissues with or without NaHS (100 μmol/L) incubation for 40 minutes were homogenized in Permeabilizatin Bufferon for 10 minutes on ice with agitation. The cell lysate was centrifuged at 16 000 g at 4°C for 15 minutes, and the supernatant was saved as cytosolic protein. The pellets were resuspended in Solubilization Buffer at 4°C for 30 minutes with constant shaking and were then centrifuged at 16 000 g at 4°C for 15 minutes. The resulting supernatants were collected as membrane fraction.

For immunobloting of PKC proteins, 70 μg of cytosolic and 100 μg of membrane proteins were separated on 8% SDS‐PAGE and transferred by electrophoresis onto an equilibrated polyvinylidene difluoride membrane. Blots were probed with primary antibodies against PKC α (GeneTex), PKC ε (Abcam), GAPDH and secondary antibodies conjugated with horseradish peroxidase (HRP). Bound antibodies were detected with an enhanced chemiluminescence detection system and analysed with AlphaEase FC software. All targeted bands were normalized to GAPDH to confirm equal protein loading.

### 2.5 Protein kinase C activity assay

Protein kinase C activity was assayed using PKC Kinase Activity Assay Kit (Abcam) as manufacturer's instructions. Briefly, total proteins from LA tissues with or without NaHS (100 μmol/L) incubation for 40 minutes were added to PKC substrate coated wells of a 96‐well microtitre plate, and reactions were initiated by adding ATP. The phosphorylated substrates were recognized by a phosphor‐PKC substrate‐specific antibody and a secondary antibody conjugated with HRP after being reacted at 30°C for 90 minutes. Bound antibodies were detected with TMB substrate, and the absorbance was measured at OD450 nm. Relative kinase activity was calculated from standard curve and normalized to individual control.

### 2.6 Measurement of intracellular reactive oxygen species

Pulmonary vein cardiomyocytes were treated with NaHS (100 μmol/L) for 40 minutes, and the reactive oxygen species (ROS) sensitive fluorescent probe CellROX Deep Red reagent (5 μmol/L, Life Technologies) was added 30 minutes before the end of the treatment. The fluorescent signals were detected on a laser scanning confocal system (Zeiss LSM 510, Carl Zeiss) equipped with the inverted microscope (Axiovert 100, Carl Zeiss) using a 60 × 1.4 numerical aperture oil immersion objective as described previously.^32^ Fluorescent images were analysed using Image‐Pro Plus 6.0 and SigmaPlot 12.3 software.

### 2.7 Statistical analyses

All continuous variables are expressed as mean ± standard error of mean. One‐way repeated‐measures analysis of variance followed by Bonferroni's analysis was used to compare the differences in PVs, SANs and LA before and after drug administration. The chi‐square analysis with Fisher's exact test was used to compare the incidences of DADs and burst firing in PVs and SANs before and after drug administration. *P* < .05 was considered statistically significant. Statistical analysis was performed using SigmaPlot 12 (Systat software).

## 3 RESULTS

### 3.1 Effects of H_2_S on the electrical activity of PVs, and SANs

**Figure 1:**
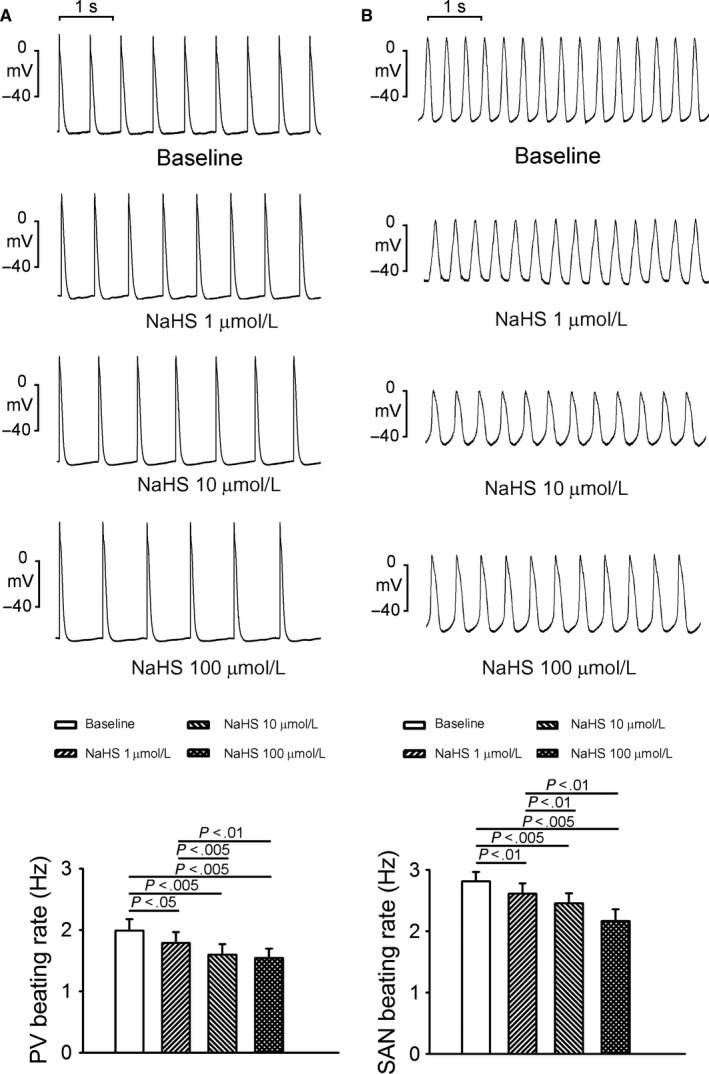
Effects of NaHS on the spontaneous activity of pulmonary veins (PVs) and sinoatrial nodes (SANs). Representative recordings and average data of beating rates in PVs (n = 10, panel A) and SANs (n = 9, panel B) before and after superfusion with different concentrations of NaHS (1, 10, and 100 μmol/L)

**Figure 2:**
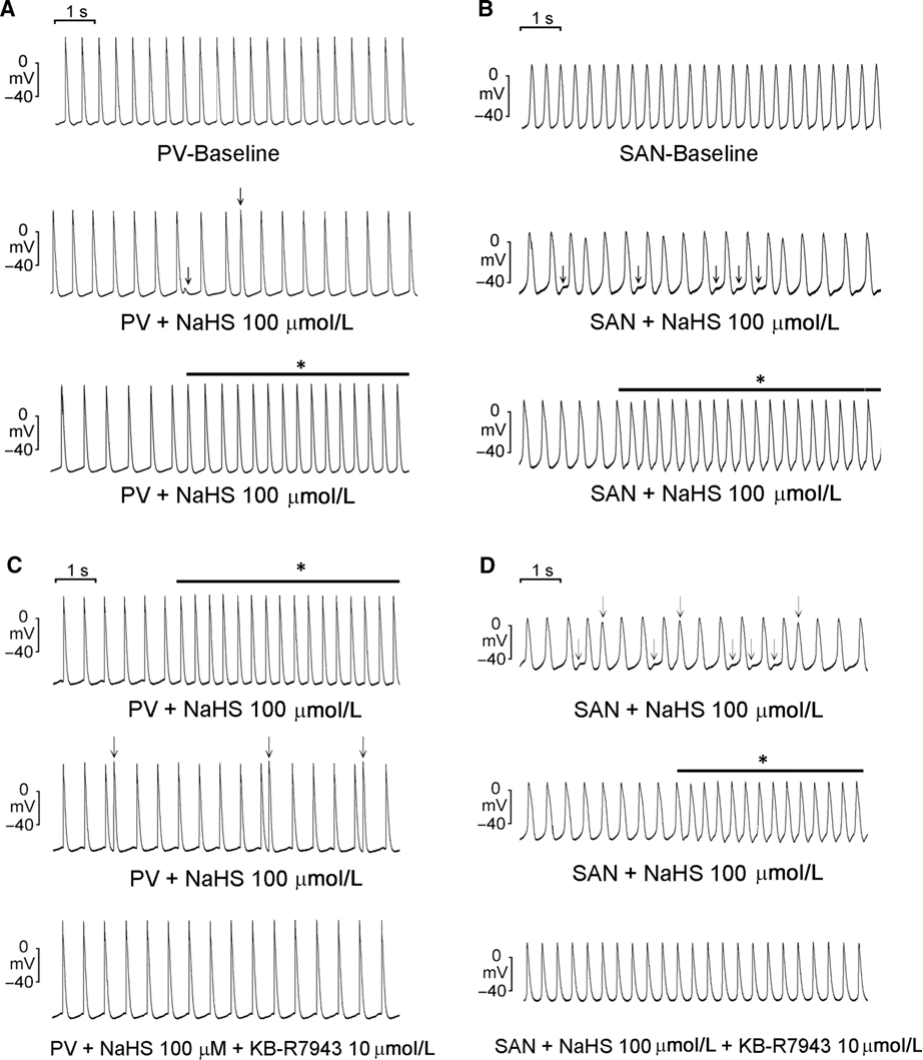
NaHS‐induced triggered activity or burst firing in pulmonary vein (PV) and sinoatrial node (SAN) preparations. A, Representative recordings of delayed after depolarization (↓) and burst firing (*) in PV preparations superfused with NaHS (100 μmol/L). B, Representative recordings of delayed after depolarization (↓) and burst firing (*) in SAN preparations superfused with NaHS (100 μmol/L). C, KB‐R7943 (10 μmol/L) suppressed NaHS (100 μmol/L)‐induced burst firing (*) and delayed afterdepolarization (↓) in PVs. D, KB‐R7943 (10 μmol/L) suppressed NaHS (100 μmol/L)‐induced delayed afterdepolarization (↓) and burst firing (*) in SANs

As shown in Figure 1A, NaHS (1, 10, and 100 μmol/L) significantly reduced the PV beating rates in a concentration‐dependent manner. However, as shown in Figure 2A, NaHS (1, 10 and 100 μmol/L) induced the occurrences of DADs in 6 PVs (60% vs 0% at baseline, *P* < .05) and induced burst firing in 5 PVs (50% vs 0% at baseline, *P* < .05). Similarly, NaHS (1, 10, and 100 μmol/L) concentration dependently reduced SAN beating rates (Figure 1B). Furthermore, NaHS (1, 10, and 100 μmol/L) induced the occurrences of DADs in 3 SANs (33% vs 0% at baseline, *P* > .05) and burst firing in 2 SANs (22% vs 0% at baseline, *P* > .05; Figure 2B). NaHS reduced SAN and PV beating rates to a similar extent (22% vs 23%, *P* > .05) but induced higher arrhythmogenicity in PVs than in SANs. Figures 2C,D show the effects of KB‐R7943 on NaHS‐induced PV and SAN arrhythmogenesis. In 6 of PVs with NaHS‐induced triggered activity, KB‐R7943 (10 μmol/L) reduced the occurrences of triggered DADs (100% vs 0%, *P* < .05) but did not change PV beating rates. Moreover, in 3 PVs with NaHS‐induced burst firing, KB‐R7943 reduced the occurrences of burst firing (100% vs 0%, *P* > .05). Similarly, in 3 SANs with NaHS‐induced triggered activity, KB‐R7943 reduced the occurrences of the triggered DADs (100% vs 0%, *P* > .05). In 2 SANs with NaHS‐induced burst firing, KB‐R7943 reduced the occurrence of burst firing (100% vs 0%, *P* > .05) but did not change SAN beating rates.

**Figure 3:**
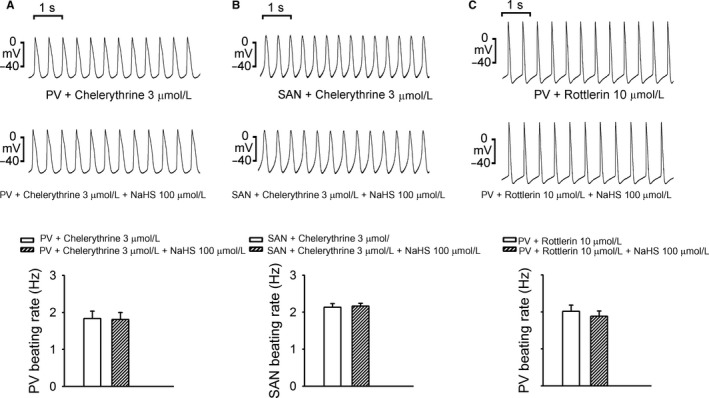
Effects of chelerythrine and rottlerin on NaHS‐induced arrhythmogenesis. A, In PVs (n = 6) pretreated with chelerythrine (3 μmol/L), NaHS (100 μmol/L) did not induce arrhythmogenesis or beating rate changes. B, In SANs (n = 6) pretreated with chelerythrine (3 μmol/L), NaHS (100 μmol/L) did not induce arrhythmogenesis or beating rate changes. C, In PVs (n = 6) pretreated with rottlerin (10 μmol/L), NaHS (100 μmol/L) did not induce arrhythmogenesis or beating rate changes

As shown in Figure 3, in the presence of chelerythrine (3 μmol/L), NaHS (100 μmol/L) did not change the beating rates or induce triggered activity and burst firing in PVs and SANs. Similarly, NaHS (100 μmol/L) did not change the beating rates, triggered activity and burst firing in PVs in the presence of rottlerin (10 μmol/L).

### 3.2 Effects of H_2_S on atrial electrical activity

**Figure 4:**
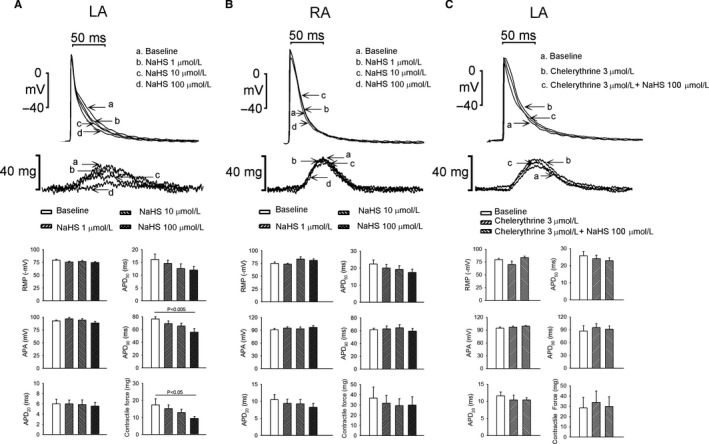
Effects of NaHS interacted with PKC inhibitors on action potential morphology and contractility of the left atrial (LA) and right atrial (RA) preparations. A, Superimposed tracings, contractility and average data of the action potential parameters in the LA preparations (n = 7) before and after superfusion with different concentrations of NaHS (1, 10 and 100 μmol/L). B, Superimposed tracings, contractility and average data of the action potential parameters in the RA preparations (n = 8) before and after superfusion with different concentrations of NaHS (1, 10, and 100 μmol/L). C, Superimposed tracings, contractility and average data of the action potential parameters in LA preparations (n = 6) pretreated with chelerythrine (3 μmol/L) and after superfusion with NaHS (100 μmol/L)

NaHS at 100 μmol/L, but not at 1 and 10 μmol/L, significantly shortened APD_90_ and reduced the contractility of the LA (Figure 4A). However, NaHS did not change the APA, RMP, APD_20_, APD_50_, APD_90_ and contractility of the RA (Figure 4B). In LA tissues pretreated with chelerythrine (3 μmol/L), NaHS (100 μmol/L) did not shorten APD_90_ or reduce contractility (Figure 4C).

### 3.3 Effects of H_2_S on I_KATP_ and I_NCX_

**Figure 5:**
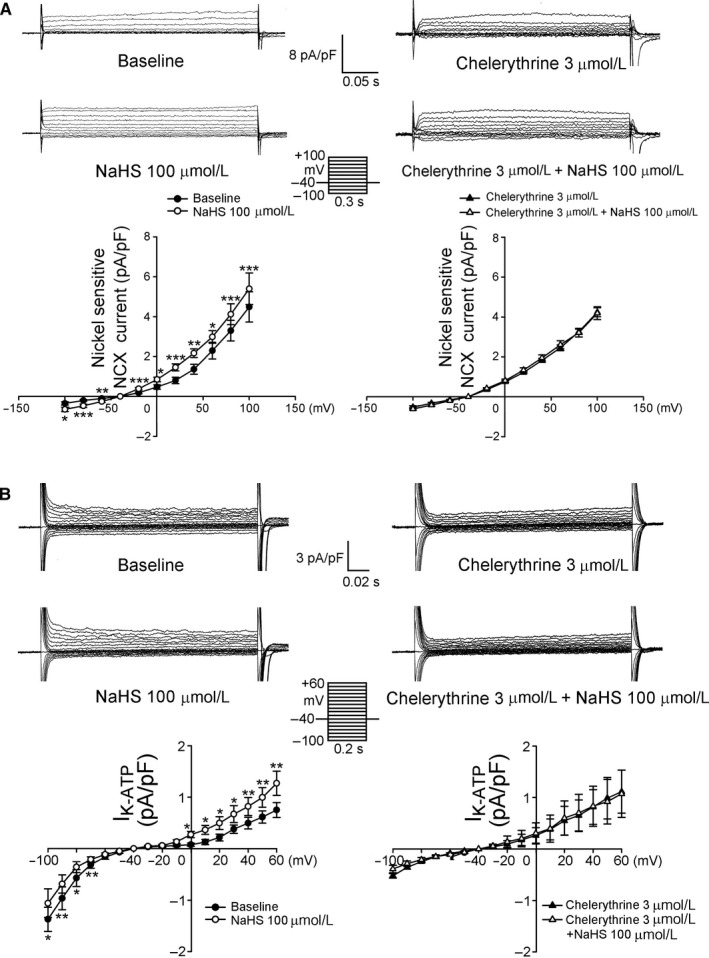
Effects of NaHS on I_NCX_ and I_KATP_ in PV cardiomyocytes. A, The tracings and current‐voltage relationship of I_NCX_ in PV cardiomyocytes before and after NaHS (100 μmol/L) with (n = 8) and without (n = 9) chelerythrine (3 μmol/L). B, The tracings and current‐voltage relationship of I_KATP_ in PV cardiomyocytes before and after NaHS (100 μmol/L) with (n = 8) and without (n = 7) chelerythrine (3 μmol/L). The insets in the current traces show the various clamp protocols. *P < .05, **P < .01, ***P < .005 vs baseline

We investigated the effects of NaHS on I_KATP_ and I_NCX_ in isolated single PV and atrial cardiomyocyte. As shown in Figure 5, NaHS (100 μmol/L) significantly increased the I_KATP_ and the forward mode of the I_NCX_. However, in the presence of chelerythrine (3 μmol/L), NaHS did not change the I_KATP_ and I_NCX_ in PV cardiomyocytes.

**Figure 6:**
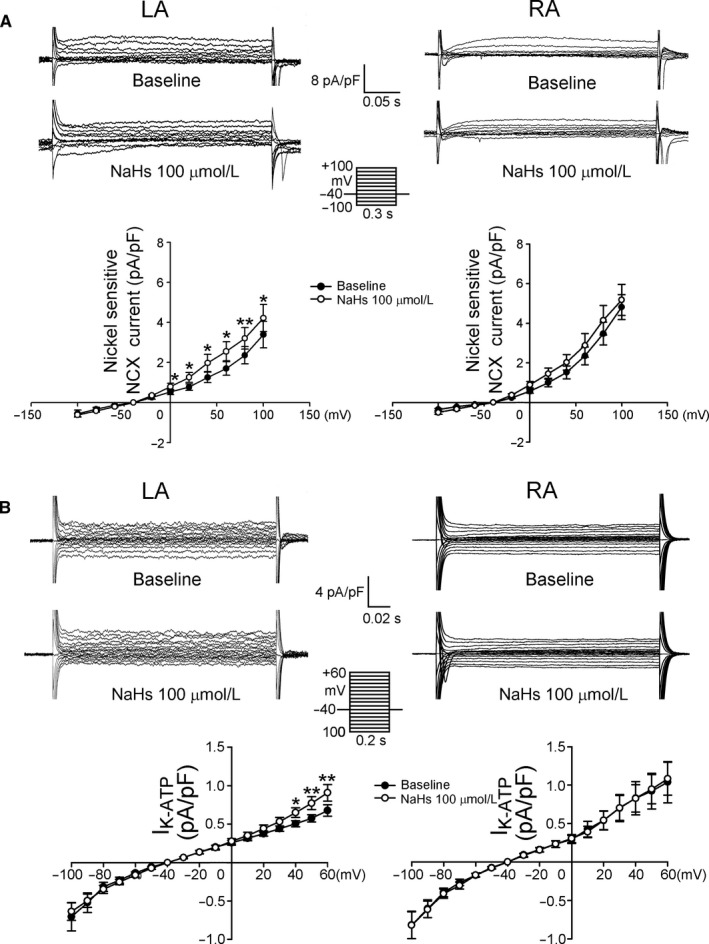
Effects of NaHS on I_NCX_ and I_KATP_ in atrial cardiomyocytes. A, The tracings and current‐voltage relationship of I_NCX_ in left atrial (LA, n = 8) and right atrial (RA, n = 9) cardiomyocytes before and after NaHS (100 μmol/L). B, The tracings and current‐voltage relationship of I_KATP_ in LA (n = 9) and RA (n = 8) cardiomyocytes before and after NaHS (100 μmol/L). The insets in the current traces show the various clamp protocols. *P < .05, **P < .01 vs baseline

We compared the effects of NaHS on I_KATP_ and I_NCX_ in LA and RA cardiomyocytes. As shown in Figure 6, NaHS significantly increased the I_NCX_ and I_KATP_ in LA cardiomyocytes, but not in RA cardiomyocytes.

### 3.4 Effect of NaHS on translocation of PKC isoforms, PKC activity and ROS

**Figure 7:**
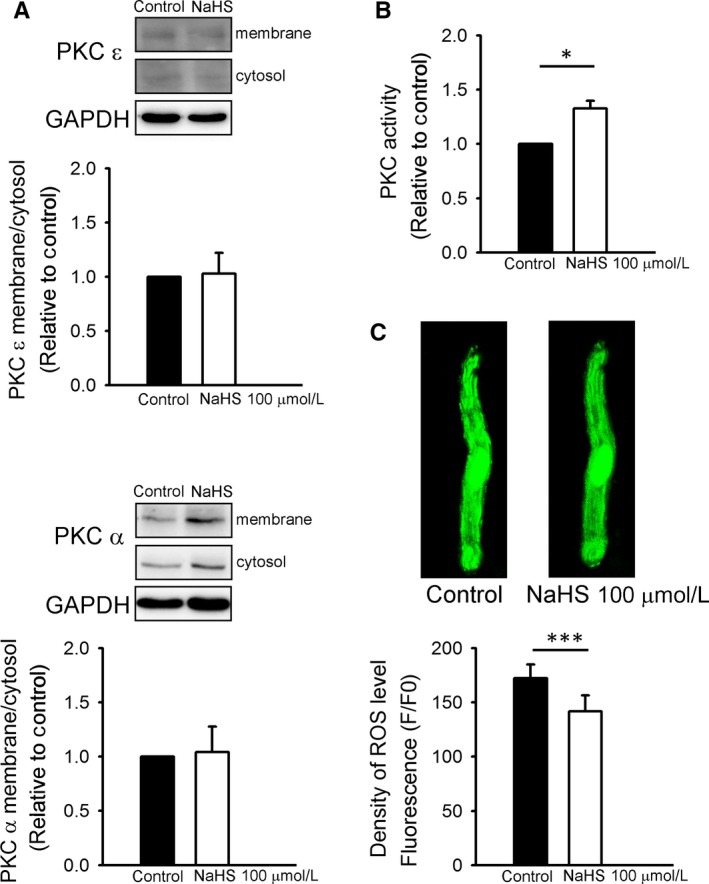
Effects of NaHS on membrane translocation of PKC isoforms, PKC activity and intracellular reactive oxygen species (ROS). A, Representative immunoblot and average data of subcellular distribution of PKC ε (n = 3) and PKC α (n = 5) from control and NaHS‐treated left atrial tissue preparations. B, The average data of relative PKC activity in control and NaHS‐treated left atrial tissue preparations (n = 3). C, An example and average data of intracellular ROS in control (n = 13) and NaHS‐treated (n = 13) PV cardiomyocytes. *P < .05, ***P < .005 vs control

As shown in Figure 7A,B, NaHS did not change the membrane to cytosol ratios of PKC α and ε in LA. However, NaHS‐treated LA had larger PKC activity than those without treatment. As shown in 7C, NaHS‐treated PV cardiomyocytes had lower ROS in cytosol than did control PV cardiomyocytes.

## 4 DISCUSSION

Air pollution is caused by multiple air pollutants, including H_2_S. This study is the first to report that H_2_S induces the occurrences of DADs and burst firing in PVs and SANs. H_2_S‐induced PKC activation is reported to play a role in regulating intracellular calcium handling by facilitating cytosolic calcium clearing through NCX channel in the development of calcium overloading and cardiomyocyte hypercontraction induced by ischaemic‐reperfusion insults.^26^ In single‐cell experiments, we revealed that H_2_S increased the forward mode of the I_NCX_ in the PV cardiomyocytes, which was attenuated by PKC inhibition. In addition, we observed that KB‐R7943 and chelerythrine suppressed NaHS‐induced PV and SAN arrhythmogenesis. These results suggest that H_2_S‐induced PKC signalling increases PV and SAN arrhythmogenesis with the activation of NCX.

Hydrogen sulphide has been reported to exert a negative chronotropic effect in SANs; this effect is inhibited by the K_ATP_ channel blocker, glibenclamide.^33^, ^34^ Similarly, this study found that H_2_S significantly reduced SAN beating rates, and this effect was attenuated by PKC inhibition. In single‐cell experiments, we revealed that NaHS increased the I_KATP_ in PV cardiomyocytes, which was attenuated by PKC inhibition. Because PKC activation is required for K_ATP_ channel opening, these results indicate that H_2_S modulates SAN function by activating PKC and K_ATP_ channels. SAN dysfunction plays an important role in AF pathophysiology^22^, ^35^ and increases PV arrhythmogenesis.^23^, ^36^ Accordingly, H_2_S may modulate SAN function and result in PV arrhythmogenesis and AF occurrence.

In the present study, NaHS increased I_KATP_ and I_NCX_ in PV cardiomyocytes, which were attenuated by chelerythrine (a selective PKC inhibitor). Additionally, chelerythrine and rottlerin (a specific PKC δ inhibitor) attenuated the arrhythmogenic effects of NaHS, thereby suggesting that PKC may mediate the effects on membrane ion currents caused by H_2_S. H_2_S diffuses through the cell membrane directly because the H_2_S molecule is very small and non‐polar. H_2_S has been shown to activate different PKC isoforms directly.^21^ Protein kinase‐catalysed phosphorylation can regulate the activity of ion channels, including the K_ATP_ and NCX channel.^37^ The activation of PKC increases the open probability of K_ATP_ channel and acts via phosphorylation of a specific, conserved threonine residue in the K_ATP_ channel.^38^ In addition, PKC directly phosphorylates NCX channel, which significantly enhances I_NCX_.^39^

Protein kinase C exists as several different isoforms and six isoforms (α, β, δ, ε, η, and ζ) were detected in hearts, among which PKC isoforms α, δ and ε are the prominent isoforms expressed in the heart. Moreover, chelerythrine is well‐known to inhibit PKC α, β1, γ and δ. Therefore, PKC α and PKC δ are more likely to be essential to NaHS‐mediated arrhythmogenesis. We found that NaHS did not induce translocation of PKC isoforms α, ε from cytosol to membrane but did increase PKC kinase activity. In the presence of rottlerin at 10 μmol/L, NaHS did not change PV electrical activity. These findings suggested that H_2_S activates PKC δ and results in its arrhythmogenesis.

The present study revealed that H_2_S differentially changed the cardiac electrophysiology of the LA and RA, whereas H_2_S significantly shortened the APD and reduced the contractility of the LA but not of the RA. These effects were attenuated by chelerythrine, suggesting that PKC signalling plays a vital role in the effects of H_2_S. The different effects of H_2_S on APD shortening in the LA and RA increase the dispersion of the APD, facilitating the maintenance of cardiac arrhythmias. Nevertheless, the mechanisms underlying the different effects of H_2_S in the LA and RA are unclear. A previous study reported that the higher expression of heat stress protein 70 in the RA may attenuate the response of the RA to the activation of the K_ATP_ channel by hypoxia and reperfusion.^40^, ^41^ Previous studies have shown that LA plays a critical role in AF genesis compared to RA. Therefore, H_2_S may have different electrophysiological effects on RA and LA cardiomyocytes. We found that NaHS significantly increased the I_NCX_ and I_KATP_ in LA cardiomyocytes but not in RA cardiomyocytes, which may result in the shortening of APD in NaHS‐treated LA.

Air pollutant is known to increase oxidative stress. We evaluated the effects of H_2_S on oxidative stress in PV cardiomyocytes by measurement of intracellular ROS using a laser scanning confocal microscope and found that NaHS‐treated PV cardiomyocytes had lower cytosol ROS than did control PV cardiomyocytes. Similarly, previous study has shown that H_2_S reduces oxidative stress in mouse model.^42^ These findings suggested that oxidative stress does not underlie the effects of H_2_S on cardiomyocytes, and H_2_S may activate PKC through its direct chemical effects, leading to the increases in I_NCX_ and I_KATP._

The effects of H_2_S has been extensively studied as an environmental pollutant.^43^, ^44^ Although H_2_S has been widely recognized as a cardioprotective agent for majority of cardiac disorders such as myocardial infarction/reperfusion injury, cardiac hypertrophy, myocardial fibrosis and heart failure,^45^ acute exposures of H_2_S may cause cardiac arrhythmia.^46^ Inhaled H_2_S induces sinus bradycardia and sinus arrest.^47^ Circulating halogen reactants cause cardiac injury by damaging important intracellular Ca^2+^ regulators.^48^ Although we do not provide a direct relationship between H_2_S and AF, our works suggest that H_2_S increases PV and SAN arrhythmogenesis and regulates atrial electrophysiology which contribute to AF.

This study should be interpreted with cautions due to the potential limitations. Air pollutants may trigger the occurrence of AF via direct and/or indirect effects on the atrial myocardium.^49^ In this study, we found that H_2_S has direct electrophysiological effects on AF substrates and triggers, supporting that H_2_S may contribute to air pollution‐induced AF at least in part. However, simply investigating H_2_S may not fully uncover the mechanisms of polluted air‐induced PV and atrial arrhythmogenesis as air pollution contains multiple pollutants in addition to H_2_S. In addition, this study found that chelerythrine or rottlerin had inhibitory effects on NaHS‐induced arrhythmogenesis, and NaHS‐treated atrium had larger PKC activity than those without treatment, suggesting that PKC pathway plays a crucial role in H_2_S‐mediated arrhythmogenesis. Nevertheless, chelerythrine is an inhibitor with multiple functions, it is also an antagonist of G‐protein‐coupled CB_1_ receptors.^50^ Studies have suggested that activation of CB_1_ receptors promotes activation of mitogen‐activated protein kinases p38 and JNK.^51^ Mitogen‐activated protein kinase is known to be functional in cardiomyocytes and is activated in response to stress, reactive oxygen species and inflammation.^52^ Rottlerin, a PKC δ inhibitor,^53^ is also an uncoupler of mitochondrial oxidative phosphorylation.^54^ Therefore, the electrophysiological data in this study did not exclude the possibility that several signalling pathways may involve the effects of H_2_S‐mediated arrhythmogenesis. The precise signalling underlying the effects of H_2_S may not be fully elucidated.

## 5 CONCLUSION

Hydrogen sulphide increases the arrhythmogenesis of PVs and SANs and differentially regulates the cardiac electrophysiology of the LA and RA. The activation of PKC signalling and increases in the I_KATP_ and I_NCX_ induced by H_2_S in PV and SAN cardiomyocytes may contribute to air pollution‐induced AF.

## CONFLICTS OF INTEREST

The authors have no conflict of interest to disclose.
